# The Detection of Pest Contaminants in Chocolate Using Visible-Near-Infrared Single-Pixel Imaging Technology

**DOI:** 10.3390/foods14020206

**Published:** 2025-01-10

**Authors:** Hidemasa Taketoshi, Tetsuya Inagaki, Satoru Tsuchikawa, Te Ma

**Affiliations:** Graduate School of Bioagricultural Sciences, Nagoya University, Furo-Cho, Chikusa, Nagoya 464-8601, Japan; taketoshi.hidemasa.x4@s.mail.nagoya-u.ac.jp (H.T.); inatetsu@agr.nagoya-u.ac.jp (T.I.); st3842@agr.nagoya-u.ac.jp (S.T.)

**Keywords:** food, contamination, insect, digital micro-mirror device, principial component analysis, image reconstruction algorithm

## Abstract

Food safety is gaining increasing attention worldwide. Currently, low-density organic foreign objects such as insects are extremely challenging to detect using conventional metal detectors and X-ray inspection systems. This study aimed to develop a visible-near-infrared single-pixel imaging (Vis-NIR-SPI) method to detect small insects inside food. The advantages of Vis-NIR light include its ability to analyze samples non-destructively and measure multiple components simultaneously and quickly, while SPI is robust against dark noise, high scattering, and high equipment costs. The experimental results demonstrated that (1) the newly designed system effectively reduces scattering effects from the highly scattering sample (intralipid 20%), allowing for the capture of information beyond the capabilities of a charge-coupled-device camera; (2) insects positioned behind ham and bread were readily detectable using the imaging reconstruction algorithm; and (3) even for chocolate samples with very high light absorption, only 1 uncontaminated sample out of 100 was mistakenly classified as contaminated, yielding an overall accuracy of 99%. This high level of accuracy underscores the potential of the Vis-NIR-SPI method to provide reliable detection while maintaining sample integrity. Furthermore, this method is cost-effective, offering a practical and efficient solution to improve quality control processes and consumer trust in the food industry.

## 1. Introduction

Food hygiene management has been gaining increasing attention worldwide. Governments and the food industry in various countries are enhancing their efforts to ensure food safety by adopting hygiene management systems based on the principles of Hazard Analysis and Critical Control Points (HACCP) [1]. In 2022, in Tokyo, Japan, consumers filed 4071 complaints concerning food with the Tokyo Metropolitan Government, of which 565 (13.9%) were related to foreign object contamination. Notably, 32.7% of these reports involved insects, making them the most frequently reported foreign object [2]. Carcasses of pests and vermin may contain spoilage bacteria, pathogens, or viruses [3]. Their contamination can cause food spoilage and discoloration and may even lead to foodborne illnesses. Although not all foreign objects threaten food safety directly, they can significantly affect consumer satisfaction. The widespread use of social media has further amplified the dissemination of such complaints, accompanied by photographic evidence that heightens a sense of urgency among consumers and businesses.

In food-manufacturing environments, the primary devices used for the non-destructive detection of pest contaminants include digital cameras, metal detectors, and X-ray inspection systems. Digital cameras are primarily used to check for surface contamination. Metal detectors can detect metals, whereas X-ray systems can identify metals, plastics, and stones [4,5]. However, the detection of low-density organic foreign objects in food, such as insects, remains highly challenging. High-energy light sources, like X-rays, easily penetrate low-density organic materials, making these objects difficult to detect. Consequently, innovative non-destructive detection methods are essential for enhancing quality control in food manufacturing processes [6,7].

One such innovative approach is terahertz time-domain spectroscopy (THz-TDS), which has recently been explored for detecting low-density organic contaminants. For example, Xudong S. (2021) applied THz-TDS and imaging technologies to investigate the feasibility of detecting insect foreign bodies in finished tea products [8]. Hu, J. et al. (2021) also utilized THz spectroscopy to detect insects in powdered milk [9]. THz-TDS offers the advantage of distinguishing between different materials based on their spectral signatures, making it a promising tool for detecting low-density contaminants like insects. However, its adoption in food manufacturing is currently limited by the complexity and high cost of the equipment.

Near-infrared (NIR) light, with wavelengths ranging from 780 to 2500 nm, is commonly used for the quality analysis of agricultural products [10,11]. The advantages of NIR light include its ability to analyze samples non-destructively and measure multiple components simultaneously and quickly. By utilizing NIR light, Whitworth et al. (2010) evaluated the freshness of fish and predicted the quality of beef by identifying and separately measuring lean and fat regions. Moscetti et al. (2014) also demonstrated its use in detecting insect-infested chestnuts [12,13]. However, it presents challenges such as considerable light scattering, which can affect light transmission. Additionally, NIR spectral imaging equipment is still costly and requires a substantial initial investment [14,15]. Therefore, highly efficient, low-cost and non-destructive inspection methods are required for the evaluation of quality and quality-related attributes [16]. 

To address these challenges, we explored the use of the single-pixel imaging (SPI) method as a low-cost and highly efficient alternative for the non-destructive detection of low-density organic contaminants. Unlike conventional THz-TDS and NIR imaging methods, SPI offers a more affordable solution while maintaining the ability to detect subtle differences in material composition. SPI, also known as ghost imaging or correlation imaging, uses a sequence of structured light patterns generated by a programmable spatial light modulator to sample an object, with light intensity measured by a single-pixel detector [17,18]. By combining the detected signals with knowledge of the illumination patterns, image reconstruction can be performed using various algorithms. SPI systems offer several advantages, including low cost, and the ability to mitigate the effects of light scattering within the samples. SPI systems typically comprise a single-pixel detector and a digital micromirror device (DMD). Light from the source passes through the DMD and the sample, and the transmitted light is collected and detected using a single-pixel detector. While a single-pixel detector alone cannot capture spatial information, varying the reflection patterns of the DMD mirrors and measuring the corresponding light intensities allow spatial information to be acquired. Subsequently, the transmission image of the sample was reconstructed using the patterns displayed on the DMD and the corresponding light intensity information. This image reconstruction process has been successfully applied in SPI systems, as demonstrated by Wang et al. (2024), where the incorporation of halide perovskites into SPI enables detailed imaging in a variety of spectral regions [19].

In this study, we aimed to non-destructively detect small low-density organic contaminants, especially insects, in food products by utilizing visible-near-infrared single-pixel imaging (Vis-NIR SPI). The objectives of this study were as follows: (1) to develop a low-cost Vis-NIR SPI system integrated with a data analysis algorithm, (2) compare the characteristics of the SPI system with those of conventional charge-coupled-device (CCD)-camera-based system using representative samples, and (3) employ bandpass filters to reconstruct images across wavelength domains and apply principal component analysis (PCA) for the nondestructive detection of insect contaminants in chocolate, a material known for its strong light absorption properties.

## 2. Materials and Methods

### 2.1. Sample Preparation

#### 2.1.1. Intralipid

To compare the scattering effects on the NIR-CCD camera and SPI system, measurements were conducted using intralipid 20% (SC-215182, Santa Cruz Biotechnology, Dallas, TX, USA).

#### 2.1.2. Ham and Bread

To evaluate the potential for insect detection between the NIR-CCD camera and the SPI system, market-sourced ham and bread samples were prepared for the study, with ham samples cut to a thickness of 5 mm and bread samples to 10 mm. Insects were positioned behind each three samples for analysis.

#### 2.1.3. Chocolate

Chocolate was chosen as the main subject because it has a uniform density and because of its strong light absorbance in visible wavelength range. The samples were prepared by embedding fruit flies (Drosophilidae) of approximately 3 mm in size into the chocolate. The chocolate samples were prepared as follows: First, the chocolate was melted by heating it in a water bath to 50 °C and then cooled to 32 °C. This process, known as tempering, was performed to reduce the scattering on the chocolate surface. Next, chocolate was poured into the container to a height of 1.5 mm, and the insect was placed inside. Subsequently, more chocolate was added until the height reached 3 mm, and the insect was embedded at a height of 1.5 mm. At this point, we confirmed that the insects were hidden within the chocolate.

### 2.2. Developing SPI System

**Figure 1:**
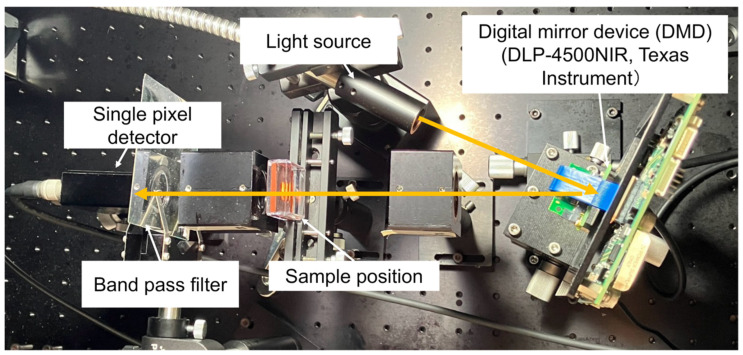
Optical path diagram of the developed single-pixel imaging system.

Figure 1 shows the optical path of the developed VIS-NIR SPI system. The SPI system was constructed using a single-pixel detector and DMD. A common scanning strategy involves using spatially resolved mask patterns and recording their intensity measurements. This method enables image formation by projecting and measuring a set of weighted patterns corresponding to the intensity measurement of each pixel. In this study, we used mask patterns based on Hadamard matrices, which are square matrices with elements of either 1 or −1, and whose rows are mutually orthogonal. If W is a Hadamard matrix and I is an identity matrix, the following equation is satisfied:

(1) $$W_{2^{k}}=\left(\begin{array}{cc} W_{2^{k-1}} & W_{2^{k-1}}\\ W_{2^{k-1}} & -W_{2^{k-1}} \end{array}\right)$$

(2) $$WW^{T}=nI$$

SPI utilizes the Walsh–Hadamard transformation, allowing simpler computations in image reconstruction and data analysis compared with other matrices. In this technique, images are acquired by combining a DMD with a single-pixel detector. The DMD consists of individually addressable micro-mirrors that can orient light transmission or blockage in ±12° directions depending on whether they are ON or OFF.

### 2.3. Building Software Control for the SPI System

We developed software with which to automatically control the DMD and detector for a single-pixel imaging setup. The setup involved converting analog signals from a single-pixel detector (C10439-08, Hamamatsu Photonics, Hamamatsu, Japan) using an analog input unit (AI-1608AY-USB, CONTEC, Osaka, Japan) to digital signals (16-bit, resolution: approximately 0.153 mV), which were then transferred to a PC. Patterns were displayed on the DMD by connecting it to a PC via HDMI, which was recognized as a second screen. Various patterns consisting of 1-bit images of 1140 × 912 pixels were displayed in full-screen mode on the second screen of the PC to the DMD. For pattern creation, displayed on the DMD, Python 3.7 was used. Patterns reconstructing 32 × 32 pixel images were created by considering 28 × 28 pixels as one pixel and arranging images with pseudo 32 × 32 pixels from the center of the DMD to 896 × 896 pixels. The device’s lowest controllable resolution is 32 × 32 pixels; however, it can be enhanced to resolutions of 64 × 64, 128 × 128, 256 × 256, and 512 × 512 pixels. While increasing the resolution enables the detection of smaller foreign objects, the measurements in this study were conducted at 32 × 32 pixels to reduce measurement time. At a resolution of 32 × 32 pixels, the spatial resolution was 0.2128 mm/pixel, while at 512 × 512 pixels, the resolution reached a maximum of 0.0076 mm/pixel. Software that automatically recorded the light intensity when displaying various patterns on the DMD was developed in Python 3.7 and C++.

### 2.4. The Image Reconstruction Principle in SPI

To obtain a sample image with N pixels, N masks are typically sequentially displayed on the DMD for sequential measurements. Image reconstruction involves storing the displayed patterns along with the detected values and reconstructing the image by performing simple matrix calculations on the detected data. When using randomly generated patterns without regularity, the noise effects increase, requiring more than N measurement cycles to reconstruct high-signal-to-noise (S/N) images. In this study, patterns based on orthogonal matrices, known as Hadamard matrices, were created to efficiently perform measurements during SPI. These patterns were created based on previous studies [20,21]. Randomly generated patterns necessitate pseudo-inverse matrix calculations, whereas with Hadamard matrices, which satisfy *W* × *W*^t^ = I as orthogonal matrices, inverse matrix operations are unnecessary. For image reconstruction, the formula involves storing the display patterns and detected values, and then computing the dot product of the inverse matrix of the display pattern with the detected values obtained after measurement to reconstruct the sample image. The formula for the image reconstruction is as follows:

(3) W·X=Y

(4) $$X=W^{-1}\cdot Y$$

*W* (Mask Pattern): The mask pattern (*W*) was generated using a DMD for SPI. Each pixel was assigned a value of 0 or 1, indicating whether it blocked (0) or transmitted (1) the light.

*X* (Sample): Sample (*X*) refers to the image reconstructed using SPI. The sample image was projected onto the DMD and the resulting light intensity measurements (*Y*) were used for image reconstruction.

*Y* (Light Intensity): Light intensity (*Y*) denotes the strength of the light measured by a single-pixel detector in the SPI after each mask pattern projection. The measured intensities were used to reconstruct the sample images.

The image reconstruction process involves using Hadamard matrices, which are orthogonal matrices with values of ±1. The DMD patterns were represented as 0 (blocking light) and 1 (allowing light to pass through). To create masks for DMD display, the ±1 values of the Hadamard matrix were converted: +1 became 1 (allow light through) and −1 became 0 (block light). During actual measurements, to compensate for the absence of negative light values, we measured the intensity when the mask *W* was applied and when its inverse mask *W*′ = 1 − *W* was applied. The difference between these two measurements was the differential spectrum used for image reconstruction. This method effectively reduces noise in the reconstructed images while minimizing the number of masks used for illumination.

### 2.5. Efficiency in Single-Pixel Imaging

Efficiency enhancement in SPI has been achieved by leveraging compressive sensing techniques [22,23]. Compressive sensing involves extracting useful data from a large dataset under the assumption that only a small portion of the data are useful, thereby reconstructing images by selectively sampling the observational data. This technique has been applied in medical MRI, where data acquisition is sparse and random sampling is feasible [24]. By assuming sparsity in the acquired pixel data, compressive sensing allows the reconstruction of images that closely resemble those obtained from all mask patterns, using fewer measurements than traditionally required. Compressive sensing offers benefits such as noise reduction and the completion of missing data.

In this study, software improvements were implemented to facilitate compressive sensing for enhancing the efficiency of SPI measurements. The computational formula used for compressive sensing is outlined below [25]:

(5) $${minimize}_{x}\;{\left\|\Phi x-y\right\|}_{2}^{2}+\lambda {\left\|\psi x\right\|}_{1}+\mu TV(x)$$

*Φ*: Hadamard matrix (<*N*);

*Ψ*: Redundant dictionary (>*N*);

*λ*, *μ*: Penalty parameters;

*TV*: Total variation term.

The Walsh ordering method adopted in this study changes the sequence of pattern illumination to enhance the efficiency of compressive sensing [26].

### 2.6. Investigation of Scattering Effects on SPI Image Reconstruction Using Scatter Samples

**Figure 2:**
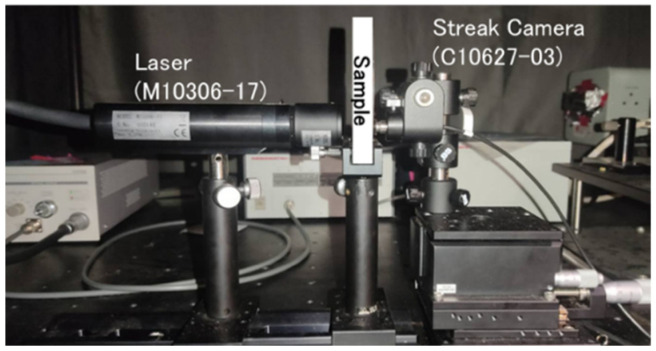
ToF-NIRS experimental device.

To evaluate the impact of light scattering in the sample on the SPI image reconstruction, the intralipid, ham and bread was measured. Intralipid is a highly scattering liquid and its scattering coefficient has been assessed in previous studies [27]. Initially, the equivalent scattering coefficient of intralipid 20% was calculated using Time-of-Flight Near-Infrared Spectroscopy (ToF-NIRS) [28]. Measurements were conducted with a scattering path length of 3 mm. The measurement setup consists of a picosecond pulsed laser (M10306-17, wavelength: 846 nm, pulse width: 70 per second, Hamamatsu Photonics Co., Hamamatsu, Japan) and a streak camera (C10627-03, time resolution: 0.3 per second, effective sensitivity wavelength range: 200 nm to 900 nm, Hamamatsu Photonics Co., Hamamatsu, Japan). Using ND filters and slits, the ND filter is set to 100% during sample measurement, and the slit is adjusted according to the observed photon quantity. Each measurement had an integration time of 60 s and was performed three times for each condition, resulting in a total measurement time of 180 s per sample. The obtained waveform was considered the transmitted pulse waveform. Figure 2 shows the ToF-NIRS setup. The optical properties were calculated using the light diffusion equation, which is a diffusion approximation of the transport equation [29]. A waveform that considers the instrument function was obtained by convolving this waveform with the reference light. This theoretical waveform was fitted to the measured waveform to calculate the absorption and equivalent scattering coefficients. Because intralipid has minimal absorption at 846 nm, the absorption coefficient was adjusted to approach zero. The obtained transmitted pulse waveform was smoothed using the Savitzky–Golay method with a quadratic polynomial, normalized such that the maximum value was 1, and fitted using the least squares method. The fitting process uses the photon observation peak as the reference, analyzing from the point where 5% of the photons are observed (5% rise) to where the photon quantity falls below 5% (5% tail) using the general programming language Python 3.7. The fitting equation was as follows:

(6) $$\begin{array}{c} T_{\left(d,t\right)}={\left(4\pi Dc\right)}^{-\frac{1}{2}}t^{-\frac{3}{2}}exp\left(-\mu_{a}ct\right)\times \\ \left\{\begin{array}{c} \left(d-Z_{0}\right)exp\left[-\frac{{\left(d-Z_{0}\right)}^{2}}{4Dct}\right]-\left(d+Z_{0}\right)exp\left[-\frac{{\left(d+Z_{0}\right)}^{2}}{4Dct}\right]\\ +\left(3d-Z_{0}\right)exp\left[-\frac{{\left(3d-Z_{0}\right)}^{2}}{4Dct}\right]-\left(3d+Z_{0}\right)exp\left[-\frac{{\left(3d+Z_{0}\right)}^{2}}{4Dct}\right] \end{array}\right\} \end{array}$$

Diffusion Coefficient (*D*): *D* = $\frac{1}{3\mu_{s}^{\prime }}$;

Penetration Depth (*Z*_0_): *Z*_0_ = $\frac{1}{\mu_{s}^{\prime }}$;

Time-Resolved Light Intensity (*T*(*d*, *t*)): T(*d*, *t*);

-(*d*): Sample thickness [mm];

-(*t*): Time [s];

-(*c*): Speed of light [mm/s];

-($\mu_{a}$): Absorption coefficient [mm^−1^];

-($\mu_{s}^{\prime }$): Reduced scattering coefficient [mm^−1^].

After calculating the equivalent scattering coefficient, the scattering effects on SPI were investigated using an NIR-CCD camera (C3077-80, Hamamatsu Photonics Co., Hamamatsu, Japan) and a single-pixel camera. The sample used for the measurements was a rectangular piece of paper (10 mm × 13 mm) with the number 25 printed on it. A 1 mm path length cell filled with 20% intralipid was placed behind the sample, and measurements were taken using both the NIR-CCD camera and the SPI system. The NIR-CCD camera utilized the halogen light source in the SPI system for these measurements. Additionally, insects were positioned behind ham and bread, replacing the previous 20% intralipid solution, with the samples placed between insects and the SPI detector for analysis.

### 2.7. Vis NIR SPI Measurement Using Bandpass Filters

To maintain the advantage of the cost-effective implementation of the SPI method, we employed three types of bandpass filters, specifically 850 nm, 910 nm, and 970 nm. These filters were chosen because of their high transmittance properties when used with chocolate samples and their relatively low costs. The filters were placed between the sample and single-pixel detector, thereby creating an optical path that could easily acquire specific spectral data from the samples. This setup allowed the reconstruction of images with a resolution of 32 × 32 pixels. Measurements were conducted for each sample using all three band-pass filters to ensure comprehensive spectral data collection. This process was repeated for 100 samples, consisting of 50 insect-contaminated and 50 uncontaminated samples. For each sample, a reference image was created using the intralipid. This reference was essential for correcting variations in the light intensity from the light source, which could otherwise have affected the accuracy of the results.

PCA was performed to extract the first principal component (PC1). The primary reason for using PCA was to emphasize the differences between chocolate- and insect-contaminated areas. The extracted PC1 data were then used to reconstruct an image, with transmittance values scaled to a range of −1 to 1. This scaling ensured that the data were normalized, thereby facilitating more accurate subsequent processing steps. Once the PC1 images were reconstructed, thresholding was applied to these images to differentiate between the chocolate and insects present within the samples. Threshold processing was implemented to achieve the highest accuracy while ensuring that foreign objects, such as insects, could always be detected. Noise processing was performed to enhance the insect detection accuracy. Specifically, noise was eliminated by removing areas smaller than five pixels, which helped reduce false positives and improved the clarity of the binarized images. The final binarized and noise-processed images were analyzed to determine the presence of insects in the chocolate samples.

## 3. Experimental Results and Discussion

### 3.1. Insect Detection Results Using Traditional X-Ray Transmission System

**Figure 3:**
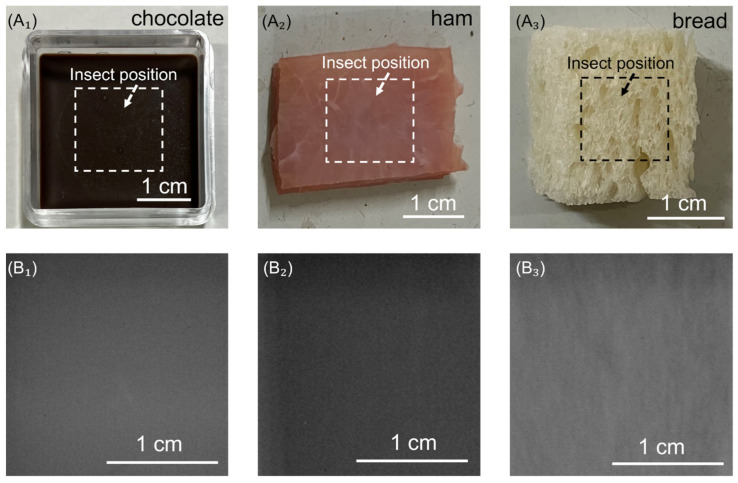
Sample contaminated with insect (**A_1_**–**A_3_**). Insect detection results using X-ray transmission system (**B_1_**–**B_3_**).

Figure 3 illustrates that small insects embedded within the chocolate, ham, and bread samples were undetected by the X-ray transmission system (MX-70eco (Mark II), mediXtec Corporation, Matsudo, Japan). SPI was introduced as an innovative technique for detecting these small insects, as traditional metal detectors are well known to be relatively ineffective in identifying them.

### 3.2. Investigation of Scattering Effects on SPI Image Reconstruction

**Figure 4:**
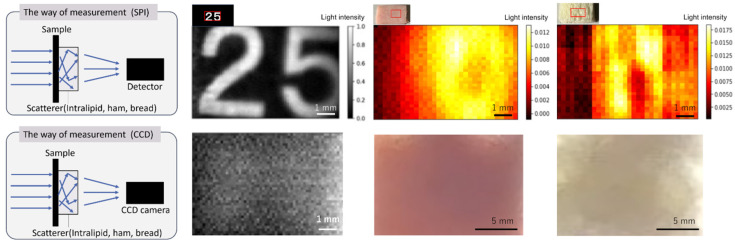
Comparison of detection results between the CCD camera and the SPI camera.

Four filters were installed to avoid the saturation of the detection device. Owing to the difference in refractive indices, causing the reference waveform to be delayed compared with some of the sample transmission pulse waveforms. Our result yielded a coefficient of 12.58 mm^−1^ for intralipid 20%. The measurement results of the NIR-CCD and SP cameras are shown in Figure 4. The measurement results when a 1 mm path length cell filled with Intralipid 20% was placed between the sample and the detector showed that the NIR-CCD camera could not distinguish number 25; in contrast, the SPI camera could still distinguish number 25, even with the scattering medium behind the sample. Even when an insect was placed behind the actual food items, ham and bread, the NIR-CCD camera could not identify the insect, but the SP camera could identify the insect (All three measured samples demonstrated consistent results). This preliminary experiment indicated that the SPI method can mitigate the scattering effects behind the sample, enabling the acquisition of information that the CCD camera cannot obtain. In this study, by utilizing the differential spectrum from the inverted masks, we successfully obtained images with low noise levels while minimizing the number of illuminated masks. Specifically, to reconstruct an image of size M × M (totaling N pixels), approximately 2N measurements are required. SPI benefits from the ability to enhance irradiation speed using a DMD, thereby complementing spatial information that cannot be detected or maintained solely by a single-pixel detector. Another advantage is the capability to visualize a wide range of wavelengths at a high resolution and low cost by varying the detection wavelength of the single-pixel detector. This enabled prolonged image capture with minimal light and a high resolution. Furthermore, the SPI exhibited robustness against dark noise, high sensitivity, and a rapid response. Given these attributes, SPI is expected to play a significant role in advancing optical imaging technologies [30,31].

### 3.3. Detection of Insects in Chocolate Using the SPI System

**Figure 5:**
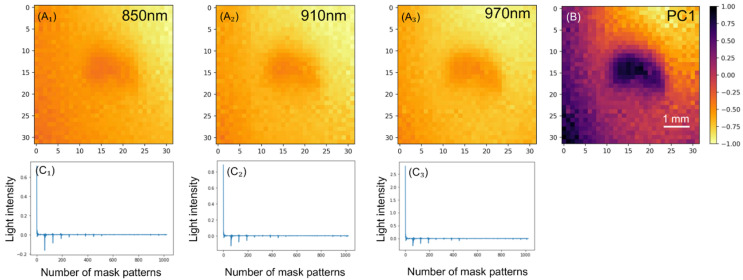
Spectroscopic images (**A₁**–**A₃**), their PC1 image (**B**), and the amount of transmitted light in each mask pattern (**C₁**–**C₃**).

Figure 5A shows the reconstructed images of the three spectral bands and Figure 5B shows the PC1 image of a representative sample. In these images, bright areas indicate higher transmittance, whereas dark areas indicate lower transmittance. The scale bar values represent the level of absorbance, with higher absorbance values corresponding to darker regions of the images. In the central area of the spectral images, there is a relatively dark region, which indicates the presence of low-density organic matter presumed to be insects. In contrast, the outer areas, which consisted of uncontaminated chocolate, exhibited a relatively higher transmittance. This clear difference in transmittance allowed the identification of insect contamination. The presence of insects was more pronounced in PC1. This enhanced contrast in the PC1 image demonstrates the effectiveness of PCA in distinguishing between different components within the samples. Figure 5C shows the signal intensity for each mask pattern displayed by the DMD. The signal intensity values indicated the amount of information captured for each mask pattern, with larger values corresponding to greater information content.

**Figure 6:**
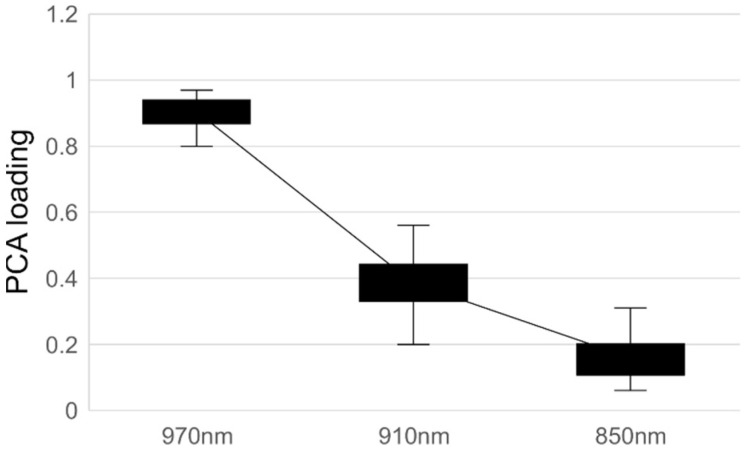
PCA loading of spectral information at 970 nm, 910 nm, and 850 nm.

Among the bandpass filters used in this experiment, the 970 nm filter was particularly useful for observing water absorption. The results indicated that the difference in transmittance between insect-contaminated and uncontaminated areas increased at longer wavelengths. This is attributed to the lower absorbance of chocolate at longer wavelengths and the higher absorption of the 970 nm wavelength by the bodily fluids of insects, while the PC loading also indicates that this wavelength is particularly effective for detecting insect contamination (Figure 6).

**Figure 7:**
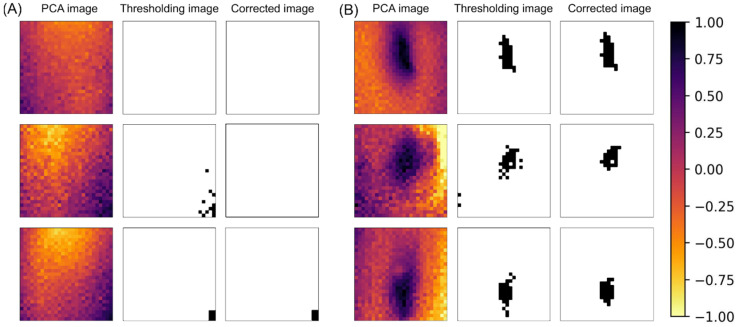
Thresholding image and corrected image of no-insect sample (**A**) and insect-contaminated sample (**B**).

Figure 7 shows the results of binarizing the PC1 images to objectively determine the presence of insects. A threshold value of 0.7 was set to evaluate the presence of insects in all 100 samples. Figure 7A shows a binarized image of an uncontaminated chocolate sample, and Figure 7B shows an example of an insect-contaminated chocolate sample. In these figures, the leftmost images are the original PC1 images, the middle images are the binarized images, and the rightmost images are the binarized images after noise processing. In the binarized and noise-processed images, completely white areas were considered uncontaminated, whereas black areas indicated the presence of insects. This analysis provides a clear and objective method for detecting insect contamination. 

This device detects foreign objects inside chocolate by receiving and calculating transmitted light intensity. By illuminating the surface of the chocolate with uniform light, differences in transmitted light intensity are minimized. However, in the sample that resulted in false positive, the chocolate was positioned at an angle, preventing uniform illumination and causing relatively large differences in transmitted light intensity compared to when uniform illumination was applied. Fixing the sample position and ensuring uniform light illumination could help reduce light inconsistencies. Nevertheless, the high accuracy achieved in this study highlights the potential of the SPI method for the non-destructive detection of insect contamination in chocolate samples.

Currently, the measurement time of 3 min per sample poses a limitation for food production lines. To overcome this, ongoing software development is focused on achieving near-real-time measurements. Through constant effort, the SPI system’s affordability, speed, and high detection performance will be fully leveraged, and significantly improving its operational efficiency. Furthermore, the development of a portable SPI device is also envisioned. Portable SPI systems have the potential to expand the application of this method to enhance food safety and quality control measures across the food supply chain.

## 4. Conclusions

In this study, we successfully demonstrate the application of SPI in conjunction with Vis-NIR light for the non-destructive detection of small, low-density organic contaminants, specifically insects, in chocolate samples. The experimental results revealed that the SPI method, particularly when employing the 970 nm bandpass filter, could effectively distinguish between insect-contaminated and uncontaminated areas. This capability was attributed to the distinct absorption characteristics of chocolate and insects at this wavelength. Of the 100 tested samples, one uncontaminated sample was mistakenly identified as contaminated, resulting in an overall accuracy of 99%. This high level of accuracy is particularly significant given the non-destructive nature of the SPI method, which preserves the integrity of the samples while providing reliable detection results.

By expanding the spectral range and optimizing the processing algorithms, the SPI system can be further enhanced to detect a broader range of contaminants and improve the detection accuracy. In this study, the detected insects were identified due to their light absorption, which caused a difference in transmitted light intensity compared to the chocolate. Similarly, by selecting appropriate wavelengths using a band-pass filter, metals and plastics can also be detected through differences in transmitted light intensity. Future research should also focus on applying this methodology to other food products with different surface textures and compositions. Furthermore, exploring the integration of more advanced spectral filters and machine learning algorithms could further enhance the detection capabilities of SPI systems. The potential to non-destructively detect low-density contaminants offers a promising avenue for ensuring food safety and quality in the food industry.

## Data Availability

The original contributions presented in this study are included in the article. Further inquiries can be directed to the corresponding author.
